# Supplementary material for: Association of polypharmacy with fall-related fractures in older Taiwanese people: age- and gender-specific analyses
Source: BMJ Open. 2014 Mar 28;4(3):e004428. doi: 10.1136/bmjopen-2013-004428 (PMC3975737; doi:10.1136/bmjopen-2013-004428)
Supplement: Author's manuscript [file bmjopen-2013-004428.draft_revisions.pdf]

Association of polypharmacy with fall-related fractures in older Taiwanese people: age- and gender-specific analyses

Journal:	*BMJ Open*
Manuscript ID:	bmjopen-2013-004428
Article Type:	Research
Date Submitted by the Author:	09-Nov-2013
Complete List of Authors:	Pan, Hsueh-Hsing; Tri-Service General Hospital, Department of Nursing<br>Li, Chung-Yi; Cheng Kung University, Graduate Institute and Department of Public Health, College of MedicineNational<br>Chen, Tzeng-Ji; Taipei Veterans General Hospital, Department of Family Medicine<br>Su, Tung-Ping; Taipei Veteran General Hospital, Department of Psychiatry<br>Wang, Kwua-Yun; Taipei Veterans General Hospital, Department of Nursing
**Primary Subject Heading**:	Pharmacology and therapeutics
Secondary Subject Heading:	Epidemiology, Geriatric medicine, Nursing
Keywords:	GENERAL MEDICINE (see Internal Medicine), GERIATRIC MEDICINE, PREVENTIVE MEDICINE

SCHOLARONE™ Manuscripts

Title:

Association of polypharmacy with fall-related fractures in older Taiwanese people: age- and gender-specific analyses

Running Title:

fall-related fractures in older people with polypharmacy

Authors names and affiliations:

Hsueh-Hsing Pan,[1] Chung-Yi Li,[2,3] Tzeng-Ji Chen,[4] Tung-Ping Su,[5] Kwua-Yun Wang[6,7]

[1] Department of Nursing, Tri-Service General Hospital, Taipei, Taiwan

[2] Graduate Institute and Department of Public Health, College of Medicine, National Cheng Kung University, Tainan, Taiwan

[3] Department of Public Health, College of Public Health, China Medical University, Taichung, Taiwan

[4] Department of Family Medicine, Taipei Veterans General Hospital, Taipei, Taiwan

[5] Department of Psychiatry, Taipei Veterans General Hospital, Taipei, Taiwan

[6] Department of Nursing, Taipei Veterans General Hospital, Taipei, Taiwan

[7] School of Nursing, National Defense Medical Center, Taipei, Taiwan

For peer review only

For peer review only - http://bmjopen.bmj.com/site/about/guidelines.xhtml

Correspondence:

Address correspondence to Kwua-Yun Wang, Department of Nursing, Taipei Veterans General Hospital, No.201, Sec.2, Shipai Rd., Taipei 112, Taiwan / School of Nursing, National Defense Medical Center, No.161, Sec. 6, Minquan E. Rd., Neihu Dist., Taipei City 114, Taiwan.

Tel: 886-2-28757233 or 886-2-87923100 ext.18766

Fax: 886-2-28757664

E-mail: w6688@mail.ndmctsgh.edu.tw

Tung-Ping Su and Kwua-Yun Wang contributed equally to this work

KEY WORDS- polypharmacy; older people; case-control study; fall-related fracture

Word count: 2223

For peer review only

For peer review only - http://bmjopen.bmj.com/site/about/guidelines.xhtml

Association of polypharmacy with fall-related fractures in older Taiwanese people: age- and gender-specific analyses

ABSTRACT

Objectives: To elucidate the associations between polypharmacy and age- and gender-specific risks of admission for fall-related fractures.

Design: Nested case-control study.

Setting: This analysis was randomly selected from all elderly beneficiaries in 2007-2008, and represents some 30% of the whole older insurers using Taiwan's National Health Insurance Research Database.

Participants: We identified 5,933 cases newly admitted for fall-related fractures during 2007-2008, and 29,665 randomly controls free from fracture.

Primary and secondary outcome measures: Polypharmacy was defined as the use of these fall-related drugs of four or more medications and prescribed related to fall within a one-year period. Logistic regression models were employed to estimate the odds ratios (ORs) and related 95% confidence intervals (CIs). The interaction of polypharmacy with age and sex was assessed separately.

Results: Compared with those who consumed no medication, older people consumed 1, 2, 3, and >=4 medications were all at significantly increased odds of developing fall-related fractures, with a significant dose-gradient pattern (β= 0.2372; P for trend< 0.0001). There were significant interactions between polypharmacy and age, but no significant interactions between polypharmacy and gender. The dose-gradient

For peer review only - http://bmjopen.bmj.com/site/about/guidelines.xhtml

relationship between number of medications and risk of fall-related fracture was more obvious in women than in men (β= 0.2515 vs. β= 0.2080). Additionally, it was most evident in older people aged 65-74 years (β= 0.2715).

Conclusions: This population-based study in Taiwan confirms the link between polypharmacy and increased the risk of fall-related fractures in older people; and highlights that elderly females and older people aged 65-74 years will be the targeted subjects for further prevention from fall-related fractures caused by polypharmacy.

KEY WORDS- polypharmacy; older people; case-control study; fall-related fracture

For peer review only

Strengths and limitations of this study

- This is a population-based study providing further evidence that polypharmacy may increase fall-related fractures risk in older people, especially in the younger age groups.
- Clinicians and researchers must consider an algorithm that may effectively identify inappropriate polypharmacy prescriptions, and more attention should be paid to the younger older people to reduce the incidence of fall-related fractures caused by polypharmacy.
- We relied on the prescription records in the database, which did not provide information on how patients use medications in the real world, and the other potential risk factors of fall, such as environmental factors and living arrangement were not available from the database.

For peer review only

For peer review only - http://bmjopen.bmj.com/site/about/guidelines.xhtml

INTRODUCTION

Older people aged over than 65 years represented 10.7% of the total population in 2010 in Taiwan.[1] Falls belong to the most common events threatening the independence of older people and occur around 30 to 40 percent of community-dwelling adults older than 65 years worldwide each year.[2] The prevalence of falls strongly increased with age, and falls were more common in women than in men.[3] Serious falls can cause fractures which lead to elder prolonging length of hospital stay, being worse quality of life, increasing cost of health care and being even death.[2, 4-6]

The incidence rate of falls increases with age and peaks in those aged over 80 years.[3] Major risk factors of falls in the elderly include functional decline, musculoskeletal problems, neurologic disease, psychosocial characteristics, and medications.[2, 4, 6] In terms of medications, polypharmacy usually increased the risk of falls higher than two-fold,[2-4] and the main group of drugs were benzodiazepines, antidepressants, antipsychotics, and antiepileptics.[7] The previous studies about the role of polypharmacy for falls in the elderly were focused on the association between hip fracture and the number of medications used per day,[8] or mainly descriptive and institution-based with limited sample size and rarely focused on fall-related fractures.[9] However, few studies have examined the polypharmacy associated with fall-related drugs on the impact of age and gender difference.

In the current study, we designed a case-control study to assess the association between polypharmacy and the risk of fall-related fractures in older people. In addition to the association between polypharmacy and the risk for fall-related fractures, the age- and gender-specific stratified analyses were also performed.

For peer review only - http://bmjopen.bmj.com/site/about/guidelines.xhtml

METHODS

Source of data

Data analyzed in this study were retrieved from Taiwan's National Health Insurance (NHI) Research Database (NHIRD) supervised by the Bureau of National Health Insurance (BNHI).[10] Taiwan reformed health insurance programs into the universal NHI system in 1995, and more than 99% of residents were enrolled in this program in 2007.[10] The NHIRD contains beneficiary registration files and claim data for reimbursement, offers information and protects the privacy and confidentiality of all beneficiaries by encrypting the personal/institutional identification numbers.

This analysis was randomly selected from all elderly beneficiaries (1,102,828) in 2007-2008, and represents some 30% of the whole older insurers made by the Review Committee of the National Health Research Institutes (NHRI) who cooperates with the BNHI to manage the NHIRD. Access to the NHI data has been approved by the NHRI.

Study design and selection of cases and controls

This is a nested case-control study design. Cases were older patients (>=65 years) hospitalized between January 1, 2007 and December 31, 2008, with a discharge diagnosis codes of fracture (800 to 829) along with accidental fall (E880 to E888) based on the International Classification of Diseases, 9th revision, Clinical Modifications (ICD-9-CM) External Cause of Injury Codes (9,10). A total of 15,054 patients were identified. In order to exclude the patients who fell during hospitalization and those who encountered fractures due to causes other than fall, we limited the cases to those who also had the same diagnostic codes at the emergency

For peer review only - http://bmjopen.bmj.com/site/about/guidelines.xhtml

visits on the same date of admission (*n*=6,015). Additionally, to ensure that all cases encountered new episodes of fall-related fracture during the study period (i.e., 2007-2008), we excluded those patients who had also encountered hospitalizations for fall or fracture, or who received outpatient services for fracture in 2006 (*n*=53). Patients hospitalized for transport accidents (ICD-9 code from E800-E848) were further excluded (*n*=29), ending up with a total of 5,933 eligible cases during the two-year period (Figure 1). The index date for each case was date of his/her first-time hospitalization for fall-related facture during the study period.

The control group was randomly selected from the study cohort free from falls or fractures during these two years, with a case/control ratio of 1/5. In total, 29,665 control subjects were selected in this study. The index date for each control subject was January 1st, 2007.

Information on fall-related risk factors

Demographic data including age on the index date, gender, and urbanization level of residential area were determined from the beneficiary registry. Age was divided into three groups: 65-74, 75-84, and ≥85 years.[8] The urbanization level for each of the 365 townships in Taiwan was categorized (i.e., urban, satellite city, and rural) according to the National Statistics of Regional Standard Classification.[11] We retrieved the information on the medical history for each subject during the 1-year period (i.e., induction period) prior to the index date. The level of co-morbidity were calculated using Charlson Cormorbidity Index (CCI).[12]

The number of medications related to fall was retrieved from medication history during the 1-year period and selected with WHO Anatomical Therapeutic Chemical

For peer review only - http://bmjopen.bmj.com/site/about/guidelines.xhtml

(ATC) classification system including allmentary tract and metabolism, blood and blood forming organs, cardiovascular system, musculo-skeletal system, nervous system, and respiratory system.[7, 9] The more details were shown in Table1. Polypharmacy was defined as “the use of these fall-related drugs of four or more medications”.[3, 13]

Statistical analysis

Continuous variables were descriptively expressed as mean ± standard deviation (SD) and proportions for categorical variables. To determine whether polypharmacy is significantly associated with the risk of admission for fall-related fractures, logistic regression models were need to calculate the crude and adjusted odds ratios (ORs) and 95% confidence intervals (CIs). In advance, the researchers investigated the association of age and gender between polypharmacy with fall-related fracture admission while controlling for other covariates. All statistical analyses were performed with SAS (version 9.2; SAS Institute, Cary, NC). A P value <0.05 was considered statistically significant.

For peer review only - http://bmjopen.bmj.com/site/about/guidelines.xhtml

RESULTS

Baseline characteristics of the cases and controls

The mean age for cases and controls was 78.3±7.5 years, and 73.9±6.7 years, respectively. Female dominance was seen in cases, but not in controls. Cases were more likely than controls to live in rural areas (43.7% vs. 36.6%), CCI score ≥3 (7.4% vs. 5.0%), and polypharmacy (14.1% vs. 6.4%) (Table2).

Polypharmacy and the risk factors of admission for fall-related fractures

Table 3 shows the OR of admission for fall-related fractures before and after adjusting the other variables. Compared with subjects aged 65-74 years, the adjusted odds ratio (AOR) of fall-related fractures for cases were 2.20 (95% CI=2.07-2.36) and 4.91(95% CI=4.51-5.35) in those aged 75-84 and ≥ 85 years, respectively. There were 2.19-fold (95% CI=2.06-2.33) of admission for fall-related fractures in females compared with males. Older people living in rural areas were at significantly increased risk of admission (AOR=1.31, 95% CI=1.22-1.40). Compared with a CCI score of 0, the risk of admission for fall-related fractures increased with CCI score, especially in those with scores ≥3 (AOR=1.35, 95% CI=1.19-1.54). In addition, there was a 2.71-fold of admission among patients who used polypharmacy (≥ 4 medications), as opposed to those used 0 medication.

Age- and gender-specific for polypharmacy and admission for fall-related fractures

Table 4 shows AOR of admission for fall-related fractures in relation to number of medications according to age and gender. There was a significant interaction between

For peer review only - http://bmjopen.bmj.com/site/about/guidelines.xhtml

number of medications and age. The significant dose-gradient relationship was noted only for those aged 65-74 years and 75-84 years, obviously in aged 65-74 years (β= 0.2715; P for trend< 0.0001). Compared with subjects who used 0 medication, subjects who used polypharmacy the AOR of admission for fall-related fractures increased to 3.23 and 2.79 for those aged 65-74 years and 75-84 years, respectively. Compared with those who used 0 medication, the risk of admission for fall-related fractures increased with the number of medications in both men and women, and there was also a significant dose-gradient relationship. The AOR associated with polypharmacy was 2.38 for men, which was lower than that for women (AOR, 2.86).

For peer review only - http://bmjopen.bmj.com/site/about/guidelines.xhtml

DISCUSSION

This study used a large population-based datasets to identify the relationship of polypharmacy and fall-related fractures in older people. The major findings were as follows: age, female, living in rural areas, comobidities and number of medications were likely to have a higher risk of admission for fall-related fractures; an increasing number of medications related to fall is independently and positively associated with the risk of admission for fall-related fractures, especially in those younger older people but not obvious in those aged ≥ 85 years; the risk of admission for fall-related fractures increased with the number of medications in both men and women, and there was also a significant dose-gradient relationship.

Our findings were similar to many previous studies that risk factors for falls are increasing with age,[4, 14, 15] and polypharmacy.[3, 4, 13, 16, 17] It is likely that older people with advanced ages tended to have higher comorbidities, lack self-sufficiency, frail, and more severe form of osteoporosis, leading to an increased risk of frequent fall and more comminuted fractures.[18, 19] Ziere et al.(2006) conducted a cross-sectional study of nearly 7 thousands individuals aged ≥ 55 years and noted that falls were more common in women than in men. A systematic review which focused on the risk factors for falls in older people also showed that women at an advanced age were a predictor for falls.[20] Our study indicated that women were more likely to suffer from fall-related fractures than men. A possible explanation was that Asian women were at a high risk of osteoporosis and had a higher incidence of hip fractures than men.[21] Older people living in rural areas were likely to have a higher risk of admission for fall-related fractures. Yiannakoulias et al.[22] investigated the relationship between geographic locations and fall injuries and the study found that the surrounding rural

For peer review only - http://bmjopen.bmj.com/site/about/guidelines.xhtml
For peer review only

regions and smaller communities had a more moderate fall incidence. A survey of falls in Taiwan indicated that most of the fall events took place outdoors, including streets sidewalks, farmlands, and mountain areas; and those who experienced fall indoor suffered the episode mainly in the living room and bathroom.[23] A cross-sectional study found that urban residents had fewer medical diagnoses, better mobility, less pain, and fewer depressive symptoms compared with rural residents.[24] Therefore, disadvantageous indoor and outdoor environments experienced by the older people from rural Taiwan might explain the urbanization and fall relationship noted in our findings.

Huang et al.[17] found a statistically significant interaction between total number of medications and falls by different age groups (<65, ≥65 years of age) in a multi-ethnic population of type 2 diabetes patients. They found a significant increase in the risk of falls with 4-5 and 7 or more medications, and the hazard ratio (HR) were 1.45 and 2.31, respectively, compared with 0-1 prescription medications. In older patients, there was a monotonic rise in the risk of falls that became statistically significant at 6-7 (HR=1.28) or more than 7 medications (HR=1.39), compared with 0-1 prescription medications. Actually, accompanying deterioration in bone quality in older people may be the high risk group for fall-related fractures.[25, 26] In short, older people with advanced ages tend to have higher comorbidities so that the medication threshold at which fall risk begins to rise may be lower in older patients than younger patients. We divided number of medications to different group, and showed that increased risk of admission for fall-related fractures in older people who had higher use of medications, with a significant dose-gradient relationship, which further substantiates the link between polypharmacy and risk of fall-related fracture. Consequently, regular reviews of older adults' medications, minimizing the drugs

For peer review only - http://bmjopen.bmj.com/site/about/guidelines.xhtml

unless those are really necessary, may be an appropriate and costless fall prevention strategy.

This study has the following strengths. First, it was population-based and included all eligible older patients in Taiwan. Therefore, the data are highly representative and allow little room for selection bias. Second, the longitudinal data and completed reimbursement claims allow for precise medication estimation. Third, the advantage of using insurance claim datasets in clinical research is easy access to the longitudinal records for a large sample of patients. Finally, we managed to adjust, in a multivariate regression model, for a number of demographic variables and CCI scores that are considered to be risk factors for fall-related fractures. This way in research may largely reduce the play of confounding, and to point up the independent effect of polypharmacy on the risk of fall-related fractures in older patients.

Several limitations inherent with the use of administrative databases also need to be mentioned. First, we relied on the prescription records in the database, which did not provide information on how patients use medications in the real world. We also used a proxy indicator for medication use- medications purchased within the past year, not medication administration. It is possible, that in a few instances, medications were prescribed but not subsequently taken. Second, medication history was observed for only 1 year before the fall-related fractures. This may underestimate the medication-associated risk of fall-related fractures. Third, only falls related to hospitalization and hospital treatment are presented. Falls which were treated in clinics, self-treated or that did not result in an injury are not included. Finally, the other potential risk factors of fall, such as environmental factors and living arrangement were not available from the NHI datasets, so that we were unable to take

For peer review only - http://bmjopen.bmj.com/site/about/guidelines.xhtml

these potential confounders in to consideration.

In summary, this population-based study provides further evidence that polypharmacy may increase fall-related fractures risk in older people, especially in the younger age groups. To further reduce the incidence of fall-related fractures caused by polypharmacy, clinicians and researchers must consider an algorithm that may effectively identify inappropriate polypharmacy prescriptions, and more attention should be paid to the younger older people.

For peer review only

For peer review only - http://bmjopen.bmj.com/site/about/guidelines.xhtml

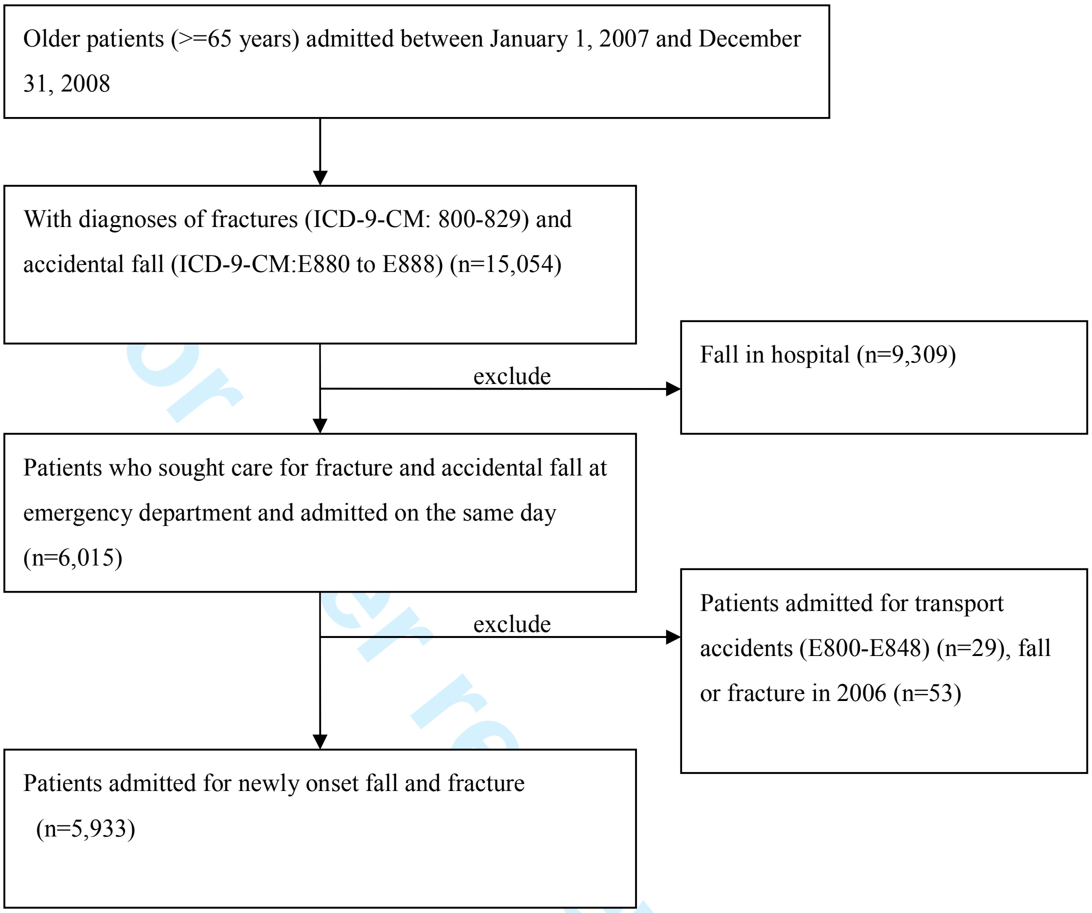

Figure 1 Flow chart of cases

For peer review only - http://bmjopen.bmj.com/site/about/guidelines.xhtml

Table 1 Medications related to fall calculated with WHO ATC classification system

Categories of medications related to fall	ATC index
**Allmentary tract and metabolism**	
Drugs for functional gastrointestinal disorders	A03
Blood glucose lowering drugs and insulin	A10A, A10B
Vitamins	A11
**Blood and blood forming organs**	
Antithrombotic agents	B01A
**Cardiovascular system**	
Cardiac glycosides and nitrates	C01A, C01DA
Antiarrhythmics	C01B
Antihypertensives	C02
Diuretics	C03
**Musculo-skeletal system**	
Non-steroidal anti-inflammatory drugs (NSAIDs)	M01A
**Nervous system**	
Opioids	N02A
Antiepileptics	N03
Antiparkinson drugs	N04
Antipsychotics	N05A
Anxiolytics	N05B
Hypnotics and sedatives	N05C
Antidepressants	N06A
**Respiratory system**	
Antihistamines	R06A

ATC, Anatomical Therapeutic Chemical

For peer review only

For peer review only - http://bmjopen.bmj.com/site/about/guidelines.xhtml

Table 2 Comparisons of sociodemographics and medications between cases and controls

	Cases (n=5,933)		Controls (n=29,665)		P value
	n	%	n	%	
Age, mean±SD	78.3	±7.5	73.9	±6.7	<0.0001
Age group, y					<0.0001
65-74	1,956	33.0	17,082	57.6	
75-84	2,671	45.0	10,270	34.6	
≥85	1,306	22.0	2,313	7.8	
Gender					<0.0001
Male	1,833	30.9	14,678	49.5	
Female	4,100	69.1	14,987	50.5	
Urbanization					<0.0001
Urban	1,930	32.5	10,934	36.9	
Satellite	1,408	23.7	7,884	26.6	
Rural	2,595	43.7	10,847	36.6	
CCI score					<0.0001
0	1,869	31.5	12,398	41.8	
1	2,595	43.7	11,982	40.4	
2	1,030	17.4	3,810	12.8	
≥3	439	7.4	1,475	5.0	
No. of medications					
0	1,008	17.0	8,113	27.4	<0.0001
1	1,551	26.1	9,204	31.0	
2	1,490	25.1	6,946	23.4	
3	1,047	17.7	3,510	13.8	
≥4	837	14.1	1,892	6.4	

CCI, Charlson comorbidity index.

For peer review only

Table 3 Crude and adjusted odds ratios and 95% confidence intervals of admission for fall-related fractures in relation to socio-demographics and medications

	Crude estimate		Adjusted estimate	
	OR	95%CI	OR	95%CI
Age group, y				
65-74	1.00	reference	1.00	reference
75-84	2.27	2.13-2.42	2.20	2.07-2.36
≥85	4.93	4.54-5.36	4.91	4.51-5.35
			β= 0.7953; P for trend< 0.0001	
Gender				
Male	1.00	reference	1.00	reference
Female	2.19	2.06-2.33	2.19	2.06-2.33
Urbanization				
Urban	1.00	reference	1.00	reference
Satellite	1.01	0.94-1.09	1.03	0.96-1.12
Rural	1.36	1.27-1.45	1.31	1.22-1.40
			β= 0.1370; P for trend< 0.0001	
CCI				
0	1.00	reference	1.00	reference
1	1.44	1.35-1.53	1.09	1.01-1.17
2	1.79	1.65-1.95	1.22	1.11-1.35
≥3	1.97	1.76-2.22	1.35	1.19-1.54
			β= 0.1008; P for trend< 0.0001	
No. of medications				
0	1.00	reference	1.00	reference
1	1.36	1.25-1.48	1.24	1.13-1.36
2	1.73	1.58-1.88	1.46	1.33-1.60
3	2.40	2.18-2.64	1.91	1.72-2.13
≥4	3.56	3.21-3.95	2.71	2.41-3.05
			β= 0.2372; P for trend< 0.0001	

CCI, Charlson comorbidity index; OR, odds ratio; CI, confidence interval.

For peer review only

Table 4 Covariate adjusted odds ratios and 95% confidence intervals of admission for fall-related fractures in relation to number of medications according to gender and age

No. of medications	Gender				Age (year)					
	Men		Women		65-74		75-84		≥85	
	AOR	95% CI	AOR	95% CI	AOR	95% CI	AOR	95% CI	AOR	95% CI
0	1.00	reference	1.00	reference	1.00	reference	1.00	reference	1.00	reference
1	1.30	1.11-1.51	1.21	1.08-1.35	1.21	1.04-1.39	1.32	1.15-1.53	1.19	0.98-1.46
2	1.46	1.24-1.72	1.45	1.29-1.63	1.44	1.24-1.68	1.64	1.42-1.90	1.16	0.94-1.44
3	1.91	1.59-2.29	1.91	1.68-2.18	1.93	1.62-2.30	2.03	1.73-2.39	1.72	1.34-2.20
≥4	2.38	1.93-2.93	2.86	2.48-3.31	3.23	2.67-3.90	2.79	2.34-3.33	1.64	1.22-2.20
	β= 0.2080; P for trend< 0.0001		β= 0.2515; P for trend< 0.0001		β= 0.2715; P for trend< 0.0001		β= 0.2453; P for trend< 0.0001		β= 0.1350; P for trend< 0.0001	

AOR, adjusted odds ratio; adjustment for age, urbanization, and Charlson index. CI, confidence interval.

For peer review only - http://bmjopen.bmj.com/site/about/guidelines.xhtml

Acknowledgements The authors would like to thank Taipei Veterans General Hospital to sponsor this study.

Contributors HH analyzed the data and wrote the manuscript. CY, TJ, TP and KY designed the study and revised the manuscript.

Funding This work was supported by a grant from Taipei Veterans General Hospital (V100C-211). The organizations that sponsored this study were not involved in the design of this study, data-collection, data processing or manuscript preparation.

Competing interests None

For peer review only

For peer review only - http://bmjopen.bmj.com/site/about/guidelines.xhtml

REFERENCES

1. Ministry of the Interior, Taiwan, 2010. *Resident population by 5-year, 10-year age group*. http://sowf.moi.gov.tw/stat/year/list.htm

2. Rao SS. Prevention of falls in older patients. *Am Fam Physician* 2005; 72: 81-8.

3. Ziere G, Dieleman JP, Hofman A*, et al.* Polypharmacy and falls in the middle age and elderly population. *Br J Clin Pharmacol* 2006; 61: 218-23.

4. Corsinovi L, Bo M, Ricauda Aimonino N*, et al.* Predictors of falls and hospitalization outcomes in elderly patients admitted to an acute geriatric unit. *Arch Gerontol Geriatr* 2009; 49: 142-5.

5. Vassallo M, Sharma JC,Allen SC. Characteristics of single fallers and recurrent fallers among hospital in-patients. *Gerontology* 2002; 48: 147-50.

6. Kannus P, Sievanen H, Palvanen M*, et al.* Prevention of falls and consequent injuries in elderly people. *Lancet* 2005; 366: 1885-93.

7. Hartikainen S, Lonnroos E,Louhivuori K. Medication as a risk factor for falls: critical systematic review. *J Gerontol A Biol Sci Med Sci* 2007; 62: 1172-81.

8. Lai SW, Liao KF, Liao CC*, et al.* Polypharmacy correlates with increased risk for hip fracture in the elderly: a population-based study. *Medicine* 2010; 89: 295-9.

9. French DD, Campbell R, Spehar A*, et al.* Outpatient medications and hip fractures in the US: a national veterans study. *Drugs Aging* 2005; 22: 877-85.

10. Lu JFR, Hsiao WC. Does universal health insurance make health care unaffordable? Lessons from Taiwan. *Health Affairs (Millwood)* 2003; 22: 77-88.

11. Directorate-General Budget , 1993. National Statistics of Regional Standard Classification Data, Taipei, Taiwan.

12. Charlson ME, Pompei P, Ales KL*, et al.* A new method of classifying prognostic

For peer review only - http://bmjopen.bmj.com/site/about/guidelines.xhtml

comorbidity in longitudinal studies: development and validation. *J Chronic Dis* 1987; 40: 373-83.

13. Baranzini F, Poloni N, Diurni M, *et al.* Polypharmacy and psychotropic drugs as risk factors for falls in long-term care setting for elderly patients in Lombardy. *Recenti Prog Med* 2009; 100: 9-16.

14. Kojima T, Akishita M, Nakamura T, *et al.* Polypharmacy as a risk for fall occurrence in geriatric outpatients. *Geriatr Gerontol Int* 2012; 12: 425-30.

15. Krauss MJ, Nguyen SL, Dunagan WC, *et al.* Circumstances of patient falls and injuries in 9 hospitals in a midwestern healthcare system. *Infection control and hospital epidemiology : the official journal of the Society of Hospital Epidemiologists of America* 2007; 28: 544-50.

16. Baranzini F, Diurni M, Ceccon F, *et al.* Fall-related injuries in a nursing home setting: is polypharmacy a risk factor? *BMC Health Serv Res* 2009; 9: 228.

17. Huang ES, Karter AJ, Danielson KK, *et al.* The association between the number of prescription medications and incident falls in a multi-ethnic population of adult type-2 diabetes patients: the diabetes and aging study. *J Gen Intern Med* 2010; 25: 141-6.

18. Chen JS, Simpson JM, March LM, *et al.* Risk factors for fracture following a fall among older people in residential care facilities in Australia. *J Am Geriatr Soc* 2008; 56: 2020-6.

19. Martinez AA, Cuenca J, Panisello JJ, *et al.* Changes in the morphology of hip fractures within a 10-year period. *Journal of bone and mineral metabolism* 2001; 19: 378-81.

20. Gama ZA,Gomez-Conesa A. [Risk factors for falls in the elderly: systematic review. *Rev Saude Publica* 2008; 42: 946-56.

21. Shao CJ, Hsieh YH, Tsai CH, *et al.* A nationwide seven-year trend of hip fractures

For peer review only - http://bmjopen.bmj.com/site/about/guidelines.xhtml

in the elderly population of Taiwan. *Bone* 2009; 44: 125-9.

22. Yiannakoulias N, Rowe BH, Svenson LW*, et al.* Zones of prevention: the geography of fall injuries in the elderly. *Social science & medicine (1982)* 2003; 57: 2065-73.

23. Chang HJ, Chang YF, Yang YC*, et al.* Epidemiological survey of falls and associated risk factors in old females in an aging community. *Taiwan Geriatrics & Gerontology* 2010; 5: 50-61.

24. Sigurdardottir AK, Arnadottir SA,Gunnarsdottir ED. Medication use among community-dwelling older Icelanders. Population-based study in urban and rural areas. *Laeknabladid* 2011; 97: 675-80.

25. Taylor AJ, Gary LC, Arora T*, et al.* Clinical and demographic factors associated with fractures among older Americans. *Osteoporos Int* 2011; 22: 1263-74.

26. Watson WL, Mitchell R. Conflicting trends in fall-related injury hospitalisations among older people: variations by injury type. *Osteoporos Int* 2011; 22: 2623-31.

For peer review only

For peer review only - http://bmjopen.bmj.com/site/about/guidelines.xhtml

STROBE 2007 (v4) Statement—Checklist of items that should be included in reports of *case-control studies*

Section/Topic	Item #	Recommendation	Reported on page #
**Title and abstract**	1	(*a*) Indicate the study's design with a commonly used term in the title or the abstract	3
		(*b*) Provide in the abstract an informative and balanced summary of what was done and what was found	3
**Introduction**			
Background/rationale	2	Explain the scientific background and rationale for the investigation being reported	6
Objectives	3	State specific objectives, including any prespecified hypotheses	6
**Methods**			
Study design	4	Present key elements of study design early in the paper	7
Setting	5	Describe the setting, locations, and relevant dates, including periods of recruitment, exposure, follow-up, and data collection	7
Participants	6	(*a*) Give the eligibility criteria, and the sources and methods of case ascertainment and control selection. Give the rationale for the choice of cases and controls	7-8
		(*b*) For matched studies, give matching criteria and the number of controls per case	8
Variables	7	Clearly define all outcomes, exposures, predictors, potential confounders, and effect modifiers. Give diagnostic criteria, if applicable	8
Data sources/ measurement	8*	For each variable of interest, give sources of data and details of methods of assessment (measurement). Describe comparability of assessment methods if there is more than one group	7-9
Bias	9	Describe any efforts to address potential sources of bias	8-9
Study size	10	Explain how the study size was arrived at	7
Quantitative variables	11	Explain how quantitative variables were handled in the analyses. If applicable, describe which groupings were chosen and why	8-9
Statistical methods	12	(*a*) Describe all statistical methods, including those used to control for confounding	9
		(*b*) Describe any methods used to examine subgroups and interactions	9
		(*c*) Explain how missing data were addressed	9
		(*d*) If applicable, explain how matching of cases and controls was addressed	8-9
		(*e*) Describe any sensitivity analyses	9

For peer review only

**Results**			
Participants	13*	(a) Report numbers of individuals at each stage of study—eg numbers potentially eligible, examined for eligibility, confirmed eligible, included in the study, completing follow-up, and analysed	10
		(b) Give reasons for non-participation at each stage	10
		(c) Consider use of a flow diagram	16, figure 1
Descriptive data	14*	(a) Give characteristics of study participants (eg demographic, clinical, social) and information on exposures and potential confounders	10
		(b) Indicate number of participants with missing data for each variable of interest	10
Outcome data	15*	Report numbers in each exposure category, or summary measures of exposure	10-11
Main results	16	(*a*) Give unadjusted estimates and, if applicable, confounder-adjusted estimates and their precision (eg, 95% confidence interval). Make clear which confounders were adjusted for and why they were included	10-11
		(*b*) Report category boundaries when continuous variables were categorized	10-11
		(*c*) If relevant, consider translating estimates of relative risk into absolute risk for a meaningful time period	10-11
Other analyses	17	Report other analyses done—eg analyses of subgroups and interactions, and sensitivity analyses	10-11
**Discussion**			
Key results	18	Summarise key results with reference to study objectives	12
Limitations	19	Discuss limitations of the study, taking into account sources of potential bias or imprecision. Discuss both direction and magnitude of any potential bias	14
Interpretation	20	Give a cautious overall interpretation of results considering objectives, limitations, multiplicity of analyses, results from similar studies, and other relevant evidence	15
Generalisability	21	Discuss the generalisability (external validity) of the study results	15
**Other information**			
Funding	22	Give the source of funding and the role of the funders for the present study and, if applicable, for the original study on which the present article is based	21

*Give information separately for cases and controls in case-control studies and, if applicable, for exposed and unexposed groups in cohort and cross-sectional studies.

Note: An Explanation and Elaboration article discusses each checklist item and gives methodological background and published examples of transparent reporting. The STROBE checklist is best used in conjunction with this article (freely available on the Web sites of PLoS Medicine at http://www.plosmedicine.org/, Annals of Internal Medicine at http://www.annals.org/, and Epidemiology at http://www.epidem.com/). Information on the STROBE Initiative is available at www.strobe-statement.org.

For peer review only

For peer review only

Association of polypharmacy with fall-related fractures in older Taiwanese people: age- and gender-specific analyses

Journal:	*BMJ Open*
Manuscript ID:	bmjopen-2013-004428.R1
Article Type:	Research
Date Submitted by the Author:	25-Feb-2014
Complete List of Authors:	Pan, Hsueh-Hsing; Tri-Service General Hospital, Department of Nursing<br>Li, Chung-Yi; Cheng Kung University, Graduate Institute and Department of Public Health, College of MedicineNational<br>Chen, Tzeng-Ji; Taipei Veterans General Hospital, Department of Family Medicine<br>Su, Tung-Ping; Taipei Veteran General Hospital, Department of Psychiatry<br>Wang, Kwua-Yun; Taipei Veterans General Hospital, Department of Nursing
**Primary Subject Heading**:	Pharmacology and therapeutics
Secondary Subject Heading:	Epidemiology, Geriatric medicine, Nursing
Keywords:	GENERAL MEDICINE (see Internal Medicine), GERIATRIC MEDICINE, PREVENTIVE MEDICINE

SCHOLARONE™ Manuscripts

For peer review only

Title:

Association of polypharmacy with fall-related fractures in older Taiwanese people: age- and gender-specific analyses

Running Title:

fall-related fractures in older people with polypharmacy

Authors names and affiliations:

Hsueh-Hsing Pan,[1,2] Chung-Yi Li,[3,4] Tzeng-Ji Chen,[5] Tung-Ping Su,[6] Kwua-Yun Wang[2,7,8]

[1] Department of Nursing, Tri-Service General Hospital, Taipei, Taiwan

[2] School of Nursing, National Defense Medical Center, Taipei, Taiwan

[3] Graduate Institute and Department of Public Health, College of Medicine, National Cheng Kung University, Tainan, Taiwan

[4] Department of Public Health, College of Public Health, China Medical University, Taichung, Taiwan

[5] Department of Family Medicine, Taipei Veterans General Hospital, Taipei, Taiwan

[6] Department of Psychiatry, Taipei Veterans General Hospital, Taipei, Taiwan

[7] Department of Nursing, Taipei Veterans General Hospital, Taipei, Taiwan

For peer review only

For peer review only - http://bmjopen.bmj.com/site/about/guidelines.xhtml

[8] School of Nursing, National Yang-Ming University, Taipei, Taiwan

Correspondence:

Address correspondence to Kwua-Yun Wang, Department of Nursing, Taipei Veterans General Hospital, No.201, Sec.2, Shipai Rd., Taipei 112, Taiwan / School of Nursing, National Defense Medical Center, No.161, Sec. 6, Minquan E. Rd., Neihu Dist., Taipei City 114, Taiwan.

Tel: 886-2-28757233 or 886-2-87923100 ext.18766

Fax: 886-2-28757664

E-mail: w6688@mail.ndmctsgh.edu.tw

Tung-Ping Su and Kwua-Yun Wang contributed equally to this work

KEY WORDS- polypharmacy; older people; case-control study; fall-related fracture

Word count: 2223

For peer review only

Association of polypharmacy with fall-related fractures in older Taiwanese people: age- and gender-specific analyses

ABSTRACT

Objectives: To elucidate the associations between polypharmacy and age- and gender-specific risks of admission for fall-related fractures.

Design: Nested case-control study.

Setting: This analysis was randomly selected from all elderly beneficiaries in 2007-2008, and represents some 30% of the whole older insurers using Taiwan's National Health Insurance Research Database.

Participants: We identified 5,933 cases newly admitted for fall-related fractures during 2007-2008, and 29,665 randomly controls free from fracture.

Primary and secondary outcome measures: Polypharmacy was defined as the use of these fall-related drugs of four or more categories of medications and prescribed related to fall within a one-year period. Logistic regression models were employed to estimate the odds ratios (ORs) and related 95% confidence intervals (CIs). The interaction of polypharmacy with age and sex was assessed separately.

Results: Compared with those who consumed no category of medication, older people consumed 1, 2, 3, and >=4 categories of medications were all at significantly increased odds of developing fall-related fractures, with a significant dose-gradient pattern (β= 0.7953; P for trend< 0.0001). There were significant interactions between polypharmacy and age, but no significant interactions between polypharmacy and

For peer review only - http://bmjopen.bmj.com/site/about/guidelines.xhtml

gender. The dose-gradient relationship between number of medication category and risk of fall-related fracture was more obvious in women than in men (β= 0.1962 vs. β= 0.1873). Additionally, it was most evident in older people aged 75-84 years (β= 0.2338).

Conclusions: This population-based study in Taiwan confirms the link between polypharmacy and increased the risk of fall-related fractures in older people; and highlights that elderly females and older people aged 75-84 years will be the targeted subjects for further prevention from fall-related fractures caused by polypharmacy.

KEY WORDS- polypharmacy; older people; case-control study; fall-related fracture

For peer review only

Strengths and limitations of this study

- This is a population-based study providing further evidence that polypharmacy may increase fall-related fractures risk in older people, especially in the middle older age groups.
- Clinicians and researchers must consider an algorithm that may effectively identify inappropriate polypharmacy prescriptions, and more attention should be paid to the middle older people to reduce the incidence of fall-related fractures caused by polypharmacy.
- We relied on the prescription records in the database, which did not provide information on how patients use medications in the real world, and the other potential risk factors of fall, such as environmental factors and living arrangement were not available from the database.

For peer review only - http://bmjopen.bmj.com/site/about/guidelines.xhtml

INTRODUCTION

Older people aged over than 65 years represented 10.7% of the total population in 2010 in Taiwan.[1] Falls belong to the most common events threatening the independence of older people and occur around 30 to 40 percent of community-dwelling adults older than 65 years worldwide each year.[2] The prevalence of falls strongly increased with age, and falls were more common in women than in men.[3] Serious falls can cause fractures which lead to elder prolonging length of hospital stay, being worse quality of life, increasing cost of health care and being even death.[2, 4-6]

The incidence rate of falls increases with age and peaks in those aged over 80 years.[3] Major risk factors of falls in the elderly include functional decline, musculoskeletal problems, neurologic disease, psychosocial characteristics, and medications.[2, 4, 6] In terms of medications, polypharmacy usually increased the risk of falls higher than two-fold,[2-4] and the main group of drugs were benzodiazepines, antidepressants, antipsychotics, and antiepileptics.[7] The previous studies about the role of polypharmacy for falls in the elderly were focused on the association between hip fracture and the number of medications used per day,[8] or mainly descriptive and institution-based with limited sample size and rarely focused on fall-related fractures.[9] However, few studies have examined the polypharmacy associated with fall-related drugs on the impact of age and gender difference.

In the current study, we designed a case-control study and aimed to assess the cumulative effect of the selected categories of medications on the risk of fall-related fractures to assess the association between polypharmacy and the risk of fall-related fractures in older people. In addition to the association between polypharmacy and the

For peer review only - http://bmjopen.bmj.com/site/about/guidelines.xhtml

risk for fall-related fractures, the age- and gender-specific stratified analyses were also performed.

METHODS

Source of data

Data analyzed in this study were retrieved from Taiwan's National Health Insurance (NHI) Research Database (NHIRD) supervised by the Bureau of National Health Insurance (BNHI).[10] Taiwan reformed health insurance programs into the universal NHI system in 1995, and more than 99% of residents were enrolled in this program in 2007.[10] The NHIRD contains beneficiary registration files and claim data for reimbursement, offers information and protects the privacy and confidentiality of all beneficiaries by encrypting the personal/institutional identification numbers.

This analysis was randomly selected from all elderly beneficiaries (1,102,828) in 2007-2008, and represents some 30% of the whole older insurers made by the Review Committee of the National Health Research Institutes (NHRI) who cooperates with the BNHI to manage the NHIRD. Access to the NHI data has been approved by the NHRI.

Study design and selection of cases and controls

This is a nested case-control study design. Cases were older patients (>=65 years) hospitalized between January 1, 2007 and December 31, 2008, with a discharge diagnosis codes of fracture (800 to 829) along with accidental fall (E880 to E888) based on the International Classification of Diseases, 9th revision, Clinical Modifications (ICD-9-CM) External Cause of Injury Codes (9,10). A total of 15,054

For peer review only - http://bmjopen.bmj.com/site/about/guidelines.xhtml

patients were identified. In order to exclude the patients who fell during hospitalization and those who encountered fractures due to causes other than fall, we limited the cases to those who also had the same diagnostic codes at the emergency visits on the same date of admission (*n*=6,015). Additionally, to ensure that all cases encountered new episodes of fall-related fracture during the study period (i.e., 2007-2008), we excluded those patients who had also encountered hospitalizations for fall or fracture, or who received outpatient services for fracture in 2006 (*n*=53). Patients hospitalized for transport accidents (ICD-9 code from E800-E848) were further excluded (*n*=29), ending up with a total of 5,933 eligible cases during the two-year period (Figure 1). The index date for each case was date of his/her first-time hospitalization for fall-related facture during the study period.

The control group was randomly selected from the study cohort free from falls or fractures during these two years, with a case/control ratio of 1/5. In total, 29,665 control subjects were selected in this study. The index date for each control subject was January 1st, 2007.

Information on fall-related risk factors

Demographic data including age on the index date, gender, and urbanization level of residential area were determined from the beneficiary registry. Age was divided into three groups: 65-74, 75-84, and $\geq$85 years.[8] The urbanization level for each of the 365 townships in Taiwan was categorized (i.e., urban, satellite city, and rural) according to the National Statistics of Regional Standard Classification.[11] We retrieved the information on the medical history for each subject during the 1-year period (i.e., induction period) prior to the index date. The level of co-morbidity were calculated using Charlson Cormorbidity Index (CCI).[12]

For peer review only - http://bmjopen.bmj.com/site/about/guidelines.xhtml

The category of medications related to fall was counted and retrieved from medication history during the 1-year period and selected with WHO Anatomical Therapeutic Chemical (ATC) classification system including allmentary tract and metabolism, blood and blood forming organs, cardiovascular system, musculo-skeletal system, and nervous system.[7, 9] The more details were shown in Table1. Polypharmacy was defined as "the use of four or more categories of the selected fall-related medications".[3, 13]

Statistical analysis

Continuous variables were descriptively expressed as mean ± standard deviation (SD) and proportions for categorical variables. To determine whether polypharmacy is significantly associated with the risk of admission for fall-related fractures, logistic regression models were need to calculate the crude and adjusted odds ratios (ORs) and 95% confidence intervals (CIs). In advance, the researchers investigated the association of age and gender between polypharmacy with fall-related fracture admission while controlling for other covariates. All statistical analyses were performed with SAS (version 9.2; SAS Institute, Cary, NC). A *P* value <0.05 was considered statistically significant.

For peer review only - http://bmjopen.bmj.com/site/about/guidelines.xhtml

RESULTS

Baseline characteristics of the cases and controls

The mean age for cases and controls was 78.3±7.5 years, and 73.9±6.7 years, respectively. Female dominance was seen in cases, but not in controls. Cases were more likely than controls to live in rural areas (43.7% vs. 36.6%), CCI score ≥3 (7.4% vs. 5.0%), and polypharmacy (57.8% vs. 41.5%) (Table2).

Polypharmacy and the risk factors of admission for fall-related fractures

Table 3 shows the OR of admission for fall-related fractures before and after adjusting the other variables. Compared with subjects aged 65-74 years, the adjusted odds ratio (AOR) of fall-related fractures for cases were 2.23 (95% CI=2.09-2.37) and 4.94(95% CI=4.53-5.37) in those aged 75-84 and ≥ 85 years, respectively. There were 2.19-fold (95% CI=2.06-2.33) of admission for fall-related fractures in females compared with males. Older people living in rural areas were at significantly increased risk of admission (AOR=1.31, 95% CI=1.23-1.41). Compared with a CCI score of 0, the risk of admission for fall-related fractures increased with CCI score, especially in those with scores ≥3 (AOR=1.46, 95% CI=1.28-1.66). In addition, there was a 2.22-fold of admission among patients who used polypharmacy (≥ 4 categories of medications), as opposed to those used 0 category of medication.

Age- and gender-specific for polypharmacy and admission for fall-related fractures

Table 4 shows AOR of admission for fall-related fractures in relation to category of medications according to age and gender. There was a significant interaction between

For peer review only - http://bmjopen.bmj.com/site/about/guidelines.xhtml

category of medications and age. The significant dose-gradient relationship was noted for those aged 65-74 years and 75-84 years, obviously in aged 75-84 years (β= 0.2338; P for trend< 0.0001). Compared with subjects who used 0 category of medication, subjects who used polypharmacy the AOR of admission for fall-related fractures increased to 2.46 and 2.40 for those aged 65-74 years and 75-84 years, respectively. Compared with those who used 0 category of medication, the risk of admission for fall-related fractures increased with the number of medication category in both men and women, and there was also a significant dose-gradient relationship. The AOR associated with polypharmacy was 2.16 for men, which was lower than that for women (AOR, 2.23).

For peer review only

For peer review only - http://bmjopen.bmj.com/site/about/guidelines.xhtml

DISCUSSION

This study used a large population-based datasets to identify the relationship of polypharmacy and fall-related fractures in older people. The major findings were as follows: age, female, living in rural areas, comobidities and category of medications were likely to have a higher risk of admission for fall-related fractures; an increasing category of medications related to fall is independently and positively associated with the risk of admission for fall-related fractures, especially in those younger older people but not obvious in those aged ≥ 85 years; the risk of admission for fall-related fractures increased with the category of medications in both men and women, and there was also a significant dose-gradient relationship.

Our findings were similar to many previous studies that risk factors for falls are increasing with age,[4, 14, 15]and polypharmacy.[3, 4, 13, 16, 17] It is likely that older people with advanced ages tended to have higher comorbidities, lack self-sufficiency, frail, and more severe form of osteoporosis, leading to an increased risk of frequent fall and more comminuted fractures.[18, 19] Ziere et al.(2006) conducted a cross-sectional study of nearly 7 thousands individuals aged ≥ 55 years and noted that falls were more common in women than in men. A systematic review which focused on the risk factors for falls in older people also showed that women at an advanced age were a predictor for falls.[20] Our study indicated that women were more likely to suffer from fall-related fractures than men. A possible explanation was that Asian women were at a high risk of osteoporosis and had a higher incidence of hip fractures than men.[21] Older people living in rural areas were likely to have a higher risk of admission for fall-related fractures. Yiannakoulias et al.[22] investigated the relationship between geographic locations and fall injuries and the study found that the surrounding rural

For peer review only - http://bmjopen.bmj.com/site/about/guidelines.xhtml

regions and smaller communities had a more moderate fall incidence. A survey of falls in Taiwan indicated that most of the fall events took place outdoors, including streets sidewalks, farmlands, and mountain areas; and those who experienced fall indoor suffered the episode mainly in the living room and bathroom.[23] A cross-sectional study found that urban residents had fewer medical diagnoses, better mobility, less pain, and fewer depressive symptoms compared with rural residents.[24] Therefore, disadvantageous indoor and outdoor environments experienced by the older people from rural Taiwan might explain the urbanization and fall relationship noted in our findings.

Huang et al.[17] found a statistically significant interaction between total number of medications and falls by different age groups (<65, ≥65 years of age) in a multi-ethnic population of type 2 diabetes patients. They found a significant increase in the risk of falls with 4-5 and 7 or more medications, and the hazard ratio (HR) were 1.45 and 2.31, respectively, compared with 0-1 prescription medications. In older patients, there was a monotonic rise in the risk of falls that became statistically significant at 6-7 (HR=1.28) or more than 7 medications (HR=1.39), compared with 0-1 prescription medications. Actually, accompanying deterioration in bone quality in older people may be the high risk group for fall-related fractures.[25, 26] In short, older people with advanced ages tend to have higher comorbidities so that the medication threshold at which fall risk begins to rise may be lower in older patients than younger patients. We divided number of medications to different group, and showed that increased risk of admission for fall-related fractures in older people who had higher use of medications, with a significant dose-gradient relationship, which further substantiates the link between polypharmacy and risk of fall-related fracture. Consequently, regular reviews of older adults' medications, minimizing the drugs

For peer review only - http://bmjopen.bmj.com/site/about/guidelines.xhtml

unless those are really necessary, may be an appropriate and costless fall prevention strategy.

This study has the following strengths. First, it was population-based and included all eligible older patients in Taiwan. Therefore, the data are highly representative and allow little room for selection bias. Second, the longitudinal data and completed reimbursement claims allow for precise medication estimation. Third, the advantage of using insurance claim datasets in clinical research is easy access to the longitudinal records for a large sample of patients. Finally, we managed to adjust, in a multivariate regression model, for a number of demographic variables and CCI scores that are considered to be risk factors for fall-related fractures. This way in research may largely reduce the play of confounding, and to point up the independent effect of polypharmacy on the risk of fall-related fractures in older patients.

Several limitations inherent with the use of administrative databases also need to be mentioned. First, we relied on the prescription records in the database, which did not provide information on how patients use medications in the real world. We also used a proxy indicator for medication use- medications purchased within the past year, not medication administration. It is possible, that in a few instances, medications were prescribed but not subsequently taken. Second, medication history was observed for only 1 year before the fall-related fractures. This may underestimate the medication-associated risk of fall-related fractures. Third, only falls related to hospitalization and hospital treatment are presented. Falls which were treated in clinics, self-treated or that did not result in an injury are not included. Finally, the other potential risk factors of fall, such as environmental factors and living arrangement were not available from the NHI datasets, so that we were unable to take

For peer review only - http://bmjopen.bmj.com/site/about/guidelines.xhtml

these potential confounders in to consideration.

In summary, this population-based study provides further evidence that polypharmacy may increase fall-related fractures risk in older people, especially in the younger age groups. To further reduce the incidence of fall-related fractures caused by polypharmacy, clinicians and researchers must consider an algorithm that may effectively identify inappropriate polypharmacy prescriptions, and more attention should be paid to the younger older people.

For peer review only

For peer review only - http://bmjopen.bmj.com/site/about/guidelines.xhtml

Table 1 Medications related to fall calculated with WHO ATC classification system

Category of medications related to fall	ATC index
**Allmentary tract and metabolism**	
Drugs for functional gastrointestinal disorders	A03
Blood glucose lowering drugs and insulin	A10A, A10B
Vitamins	A11
**Blood and blood forming organs**	
Antithrombotic agents	B01A
**Cardiovascular system**	
Cardiac glycosides and nitrates	C01A, C01DA
Antiarrhythmics	C01B
Antihypertensives	C02
Diuretics	C03
**Musculo-skeletal system**	
Non-steroidal anti-inflammatory drugs (NSAIDs)	M01A
**Nervous system**	
Opioids	N02A
Antiepileptics	N03
Antiparkinson drugs	N04
Antipsychotics	N05A
Anxiolytics	N05B
Hypnotics and sedatives	N05C
Antidepressants	N06A

ATC, Anatomical Therapeutic Chemical

For peer review only

For peer review only - http://bmjopen.bmj.com/site/about/guidelines.xhtml

Table 2 Comparisons of sociodemographics and medications between cases and controls

	Cases (n=5,933)		Controls (n=29,665)		P value
	n	%	n	%	
Age, mean±SD	78.3	±7.5	73.9	±6.7	<0.0001
Age group, y					<0.0001
65-74	1,956	33.0	17,082	57.6	
75-84	2,671	45.0	10,270	34.6	
≥85	1,306	22.0	2,313	7.8	
Gender					<0.0001
Male	1,833	30.9	14,678	49.5	
Female	4,100	69.1	14,987	50.5	
Urbanization					<0.0001
Urban	1,930	32.5	10,934	36.9	
Satellite	1,408	23.7	7,884	26.6	
Rural	2,595	43.7	10,847	36.6	
CCI score					<0.0001
0	1,869	31.5	12,398	41.8	
1	2,595	43.7	11,982	40.4	
2	1,030	17.4	3,810	12.8	
≥3	439	7.4	1,475	5.0	
Category of medications					
0	348	5.9	3,464	11.7	<0.0001
1	523	8.8	3,984	13.4	
2	775	13.1	4,913	16.6	
3	857	14.4	4,995	16.8	
>=4	3,430	57.8	12,309	41.5	

CCI, Charlson comorbidity index.

For peer review only

Table 3 Crude and adjusted odds ratios and 95% confidence intervals of admission for fall-related fractures in relation to socio-demographics and medications

	Crude estimate		Adjusted estimate	
	OR	95%CI	OR	95%CI
Age group, y				
65-74	1.00	reference	1.00	reference
75-84	2.27	2.13-2.42	2.23	2.09-2.37
≥85	4.93	4.54-5.36	4.94	4.53-5.37
			β= 0.7953; P for trend< 0.0001	
Gender				
Male	1.00	reference	1.00	reference
Female	2.19	2.06-2.33	2.19	2.06-2.33
Urbanization				
Urban	1.00	reference	1.00	reference
Satellite	1.01	0.94-1.09	1.03	0.96-1.12
Rural	1.36	1.27-1.45	1.31	1.23-1.41
			β= 0.1370; P for trend< 0.0001	
CCI				
0	1.00	reference	1.00	reference
1	1.44	1.35-1.53	1.08	1.00-1.16
2	1.79	1.65-1.95	1.26	1.15-1.39
≥3	1.97	1.76-2.22	1.46	1.28-1.66
			β= 0.1008; P for trend< 0.0001	
Category of medications				
0	1.00	reference	1.00	reference
1	1.31	1.13-1.51	1.29	1.11-1.49
2	1.57	1.37-1.80	1.46	1.33-1.60
3	1.71	1.50-1.95	1.51	1.31-1.73
≥4	2.77	2.47-3.12	2.22	1.95-2.52
			β= 0.1943; P for trend< 0.0001	

CCI, Charlson comorbidity index; OR, odds ratio; CI, confidence interval.

For peer review only - http://bmjopen.bmj.com/site/about/guidelines.xhtml

Table 4 Covariate adjusted odds ratios and 95% confidence intervals of admission for fall-related fractures in relation to category of medications according to gender and age

Category of medications	Gender				Age (year)					
	Men		Women		65-74		75-84		≥85	
	AOR	95% CI	AOR	95% CI	AOR	95% CI	AOR	95% CI	AOR	95% CI
0	1.00	reference	1.00	reference	1.00	reference	1.00	reference	1.00	reference
1	1.31	1.03-1.67	1.26	1.05-1.52	1.49	1.17-1.90	1.11	0.86-1.43	1.34	1.00-1.81
2	1.39	1.10-1.75	1.48	1.24-1.77	1.66	1.32-2.09	1.55	1.23-1.96	1.12	0.84-1.50
3	1.55	1.23-1.96	1.47	1.24-1.75	1.62	1.28-2.04	1.71	1.36-2.14	1.13	0.84-1.51
≥4	2.16	1.75-2.68	2.23	1.90-2.62	2.46	1.98-3.05	2.40	1.95-2.97	1.69	1.30-2.20
	β= 0.1873; P for trend< 0.0001		β= 0.1962; P for trend< 0.0001		β= 0.1962; P for trend< 0.0001		β= 0.2338; P for trend< 0.0001		β= 0.1168; P for trend< 0.0001	

AOR, adjusted odds ratio; adjustment for age, urbanization, and Charlson index. CI, confidence interval.

For peer review only

Acknowledgements The authors would like to thank Taipei Veterans General Hospital to sponsor this study.

Funding This work was supported by a grant from Taipei Veterans General Hospital (V100C-211). The organizations that sponsored this study were not involved in the design of this study, data-collection, data processing or manuscript preparation.

Contributors HH analyzed the data and wrote the manuscript. CY, TJ, TP and KY designed the study and revised the manuscript.

Competing interests None declared

Data Sharing Statement: None

For peer review only

REFERENCES

1. Ministry of the Interior, Taiwan, 2010. *Resident population by 5-year, 10-year age group*. http://sowf.moi.gov.tw/stat/year/list.htm

2. Rao SS. Prevention of falls in older patients. *Am Fam Physician* 2005; 72: 81-8.

3. Ziere G, Dieleman JP, Hofman A*, et al.* Polypharmacy and falls in the middle age and elderly population. *Br J Clin Pharmacol* 2006; 61: 218-23.

4. Corsinovi L, Bo M, Ricauda Aimonino N*, et al.* Predictors of falls and hospitalization outcomes in elderly patients admitted to an acute geriatric unit. *Arch Gerontol Geriatr* 2009; 49: 142-5.

5. Vassallo M, Sharma JC,Allen SC. Characteristics of single fallers and recurrent fallers among hospital in-patients. *Gerontology* 2002; 48: 147-50.

6. Kannus P, Sievanen H, Palvanen M*, et al.* Prevention of falls and consequent injuries in elderly people. *Lancet* 2005; 366: 1885-93.

7. Hartikainen S, Lonnroos E, Louhivuori K. Medication as a risk factor for falls: critical systematic review. *J Gerontol A Biol Sci Med Sci* 2007; 62: 1172-81.

8. Lai SW, Liao KF, Liao CC*, et al.* Polypharmacy correlates with increased risk for hip fracture in the elderly: a population-based study. *Medicine* 2010; 89: 295-9.

9. French DD, Campbell R, Spehar A*, et al.* Outpatient medications and hip fractures in the US: a national veterans study. *Drugs Aging* 2005; 22: 877-85.

10. Lu JFR, Hsiao WC. Does universal health insurance make health care unaffordable? Lessons from Taiwan. *Health Affairs (Millwood)* 2003; 22: 77-88.

11. Directorate-General Budget , 1993. National Statistics of Regional Standard Classification Data, Taipei, Taiwan.

12. Charlson ME, Pompei P, Ales KL*, et al.* A new method of classifying prognostic

For peer review only - http://bmjopen.bmj.com/site/about/guidelines.xhtml

comorbidity in longitudinal studies: development and validation. *J Chronic Dis* 1987; 40: 373-83.

13. Baranzini F, Poloni N, Diurni M*, et al.* Polypharmacy and psychotropic drugs as risk factors for falls in long-term care setting for elderly patients in Lombardy. *Recenti Prog Med* 2009; 100: 9-16.

14. Kojima T, Akishita M, Nakamura T*, et al.* Polypharmacy as a risk for fall occurrence in geriatric outpatients. *Geriatr Gerontol Int* 2012; 12: 425-30.

15. Krauss MJ, Nguyen SL, Dunagan WC*, et al.* Circumstances of patient falls and injuries in 9 hospitals in a midwestern healthcare system. *Infection control and hospital epidemiology : the official journal of the Society of Hospital Epidemiologists of America* 2007; 28: 544-50.

16. Baranzini F, Diurni M, Ceccon F*, et al.* Fall-related injuries in a nursing home setting: is polypharmacy a risk factor? *BMC Health Serv Res* 2009; 9: 228.

17. Huang ES, Karter AJ, Danielson KK*, et al.* The association between the number of prescription medications and incident falls in a multi-ethnic population of adult type-2 diabetes patients: the diabetes and aging study. *J Gen Intern Med* 2010; 25: 141-6.

18. Chen JS, Simpson JM, March LM*, et al.* Risk factors for fracture following a fall among older people in residential care facilities in Australia. *J Am Geriatr Soc* 2008; 56: 2020-6.

19. Martinez AA, Cuenca J, Panisello JJ*, et al.* Changes in the morphology of hip fractures within a 10-year period. *Journal of bone and mineral metabolism* 2001; 19: 378-81.

20. Gama ZA,Gomez-Conesa A. [Risk factors for falls in the elderly: systematic review. *Rev Saude Publica* 2008; 42: 946-56.

21. Shao CJ, Hsieh YH, Tsai CH*, et al.* A nationwide seven-year trend of hip fractures

For peer review only - http://bmjopen.bmj.com/site/about/guidelines.xhtml

in the elderly population of Taiwan. *Bone* 2009; 44: 125-9.

22. Yiannakoulias N, Rowe BH, Svenson LW*, et al.* Zones of prevention: the geography of fall injuries in the elderly. *Social science & medicine (1982)* 2003; 57: 2065-73.

23. Chang HJ, Chang YF, Yang YC*, et al.* Epidemiological survey of falls and associated risk factors in old females in an aging community. *Taiwan Geriatrics & Gerontology* 2010; 5: 50-61.

24. Sigurdardottir AK, Arnadottir SA,Gunnarsdottir ED. Medication use among community-dwelling older Icelanders. Population-based study in urban and rural areas. *Laeknabladid* 2011; 97: 675-80.

25. Taylor AJ, Gary LC, Arora T*, et al.* Clinical and demographic factors associated with fractures among older Americans. *Osteoporos Int* 2011; 22: 1263-74.

26. Watson WL, Mitchell R. Conflicting trends in fall-related injury hospitalisations among older people: variations by injury type. *Osteoporos Int* 2011; 22: 2623-31.

For peer review only

For peer review only - http://bmjopen.bmj.com/site/about/guidelines.xhtml

Title:

Association of polypharmacy with fall-related fractures in older Taiwanese people: age- and gender-specific analyses

Running Title:

fall-related fractures in older people with polypharmacy

Authors names and affiliations:

Hsueh-Hsing Pan,[1] Chung-Yi Li,[2,3] Tzeng-Ji Chen,[4] Tung-Ping Su,[5] Kwua-Yun Wang[6,7]

[1] Department of Nursing, Tri-Service General Hospital, Taipei, Taiwan

[2] Graduate Institute and Department of Public Health, College of Medicine, National Cheng Kung University, Tainan, Taiwan

[3] Department of Public Health, College of Public Health, China Medical University, Taichung, Taiwan

[4] Department of Family Medicine, Taipei Veterans General Hospital, Taipei, Taiwan

[5] Department of Psychiatry, Taipei Veterans General Hospital, Taipei, Taiwan

[6] Department of Nursing, Taipei Veterans General Hospital, Taipei, Taiwan

[7] School of Nursing, National Defense Medical Center, Taipei, Taiwan

For peer review only - http://bmjopen.bmj.com/site/about/guidelines.xhtml

Correspondence:

Address correspondence to Kwua-Yun Wang, Department of Nursing, Taipei Veterans General Hospital, No.201, Sec.2, Shipai Rd., Taipei 112, Taiwan / School of Nursing, National Defense Medical Center, No.161, Sec. 6, Minquan E. Rd., Neihu Dist., Taipei City 114, Taiwan.

Tel: 886-2-28757233 or 886-2-87923100 ext.18766

Fax: 886-2-28757664

E-mail: w6688@mail.ndmctsgh.edu.tw

Tung-Ping Su and Kwua-Yun Wang contributed equally to this work

KEY WORDS- polypharmacy; older people; case-control study; fall-related fracture

Word count: 2223

For peer review only

Association of polypharmacy with fall-related fractures in older Taiwanese people: age- and gender-specific analyses

ABSTRACT

Objectives: To elucidate the associations between polypharmacy and age- and gender-specific risks of admission for fall-related fractures.

Design: Nested case-control study.

Setting: This analysis was randomly selected from all elderly beneficiaries in 2007-2008, and represents some 30% of the whole older insurers using Taiwan's National Health Insurance Research Database.

Participants: We identified 5,933 cases newly admitted for fall-related fractures during 2007-2008, and 29,665 randomly controls free from fracture.

Primary and secondary outcome measures: Polypharmacy was defined as the use of these fall-related drugs of four or more categories of medications and prescribed related to fall within a one-year period. Logistic regression models were employed to estimate the odds ratios (ORs) and related 95% confidence intervals (CIs). The interaction of polypharmacy with age and sex was assessed separately.

Results: Compared with those who consumed no category of medication, older people consumed 1, 2, 3, and >=4 categories of medications were all at significantly increased odds of developing fall-related fractures, with a significant dose-gradient pattern (β= 0.7953; P for trend< 0.0001). There were significant interactions between polypharmacy and age, but no significant interactions between polypharmacy and

For peer review only - http://bmjopen.bmj.com/site/about/guidelines.xhtml

gender. The dose-gradient relationship between number of medication category and risk of fall-related fracture was more obvious in women than in men (β= 0.1962 vs. β= 0.1873). Additionally, it was most evident in older people aged 75-84 years (β= 0.2338).

Conclusions: This population-based study in Taiwan confirms the link between polypharmacy and increased the risk of fall-related fractures in older people; and highlights that elderly females and older people aged 75-84 years will be the targeted subjects for further prevention from fall-related fractures caused by polypharmacy.

KEY WORDS- polypharmacy; older people; case-control study; fall-related fracture

For peer review only

For peer review only - http://bmjopen.bmj.com/site/about/guidelines.xhtml

Strengths and limitations of this study

- This is a population-based study providing further evidence that polypharmacy may increase fall-related fractures risk in older people, especially in the middle older age groups.
- Clinicians and researchers must consider an algorithm that may effectively identify inappropriate polypharmacy prescriptions, and more attention should be paid to the middle older people to reduce the incidence of fall-related fractures caused by polypharmacy.
- We relied on the prescription records in the database, which did not provide information on how patients use medications in the real world, and the other potential risk factors of fall, such as environmental factors and living arrangement were not available from the database.

For peer review only

For peer review only - http://bmjopen.bmj.com/site/about/guidelines.xhtml

INTRODUCTION

Older people aged over than 65 years represented 10.7% of the total population in 2010 in Taiwan.[1] Falls belong to the most common events threatening the independence of older people and occur around 30 to 40 percent of community-dwelling adults older than 65 years worldwide each year.[2] The prevalence of falls strongly increased with age, and falls were more common in women than in men.[3] Serious falls can cause fractures which lead to elder prolonging length of hospital stay, being worse quality of life, increasing cost of health care and being even death.[2, 4-6]

The incidence rate of falls increases with age and peaks in those aged over 80 years.[3] Major risk factors of falls in the elderly include functional decline, musculoskeletal problems, neurologic disease, psychosocial characteristics, and medications.[2, 4, 6] In terms of medications, polypharmacy usually increased the risk of falls higher than two-fold,[2-4] and the main group of drugs were benzodiazepines, antidepressants, antipsychotics, and antiepileptics.[7] The previous studies about the role of polypharmacy for falls in the elderly were focused on the association between hip fracture and the number of medications used per day,[8] or mainly descriptive and institution-based with limited sample size and rarely focused on fall-related fractures.[9] However, few studies have examined the polypharmacy associated with fall-related drugs on the impact of age and gender difference.

In the current study, we designed a case-control study and aimed to assess the cumulative effect of the selected categories of medications on the risk of fall-related fractures to assess the association between polypharmacy and the risk of fall-related fractures in older people. In addition to the association between polypharmacy and the

For peer review only - http://bmjopen.bmj.com/site/about/guidelines.xhtml

risk for fall-related fractures, the age- and gender-specific stratified analyses were also performed.

METHODS

Source of data

Data analyzed in this study were retrieved from Taiwan's National Health Insurance (NHI) Research Database (NHIRD) supervised by the Bureau of National Health Insurance (BNHI).[10] Taiwan reformed health insurance programs into the universal NHI system in 1995, and more than 99% of residents were enrolled in this program in 2007.[10] The NHIRD contains beneficiary registration files and claim data for reimbursement, offers information and protects the privacy and confidentiality of all beneficiaries by encrypting the personal/institutional identification numbers.

This analysis was randomly selected from all elderly beneficiaries (1,102,828) in 2007-2008, and represents some 30% of the whole older insurers made by the Review Committee of the National Health Research Institutes (NHRI) who cooperates with the BNHI to manage the NHIRD. Access to the NHI data has been approved by the NHRI.

Study design and selection of cases and controls

This is a nested case-control study design. Cases were older patients (>=65 years) hospitalized between January 1, 2007 and December 31, 2008, with a discharge diagnosis codes of fracture (800 to 829) along with accidental fall (E880 to E888) based on the International Classification of Diseases, 9th revision, Clinical Modifications (ICD-9-CM) External Cause of Injury Codes (9,10). A total of 15,054

For peer review only - http://bmjopen.bmj.com/site/about/guidelines.xhtml

patients were identified. In order to exclude the patients who fell during hospitalization and those who encountered fractures due to causes other than fall, we limited the cases to those who also had the same diagnostic codes at the emergency visits on the same date of admission (*n*=6,015). Additionally, to ensure that all cases encountered new episodes of fall-related fracture during the study period (i.e., 2007-2008), we excluded those patients who had also encountered hospitalizations for fall or fracture, or who received outpatient services for fracture in 2006 (*n*=53). Patients hospitalized for transport accidents (ICD-9 code from E800-E848) were further excluded (*n*=29), ending up with a total of 5,933 eligible cases during the two-year period (Figure 1). The index date for each case was date of his/her first-time hospitalization for fall-related facture during the study period.

The control group was randomly selected from the study cohort free from falls or fractures during these two years, with a case/control ratio of 1/5. In total, 29,665 control subjects were selected in this study. The index date for each control subject was January 1st, 2007.

Information on fall-related risk factors

Demographic data including age on the index date, gender, and urbanization level of residential area were determined from the beneficiary registry. Age was divided into three groups: 65-74, 75-84, and ≥85 years.[8] The urbanization level for each of the 365 townships in Taiwan was categorized (i.e., urban, satellite city, and rural) according to the National Statistics of Regional Standard Classification.[11] We retrieved the information on the medical history for each subject during the 1-year period (i.e., induction period) prior to the index date. The level of co-morbidity were calculated using Charlson Cormorbidity Index (CCI).[12]

For peer review only - http://bmjopen.bmj.com/site/about/guidelines.xhtml

The category of medications related to fall was counted and retrieved from medication history during the 1-year period and selected with WHO Anatomical Therapeutic Chemical (ATC) classification system including allmentary tract and metabolism, blood and blood forming organs, cardiovascular system, musculo-skeletal system, and nervous system.[7, 9] The more details were shown in Table1. Polypharmacy was defined as "the use of four or more categories of the selected fall-related medications".[3, 13]

Statistical analysis

Continuous variables were descriptively expressed as mean ± standard deviation (SD) and proportions for categorical variables. To determine whether polypharmacy is significantly associated with the risk of admission for fall-related fractures, logistic regression models were need to calculate the crude and adjusted odds ratios (ORs) and 95% confidence intervals (CIs). In advance, the researchers investigated the association of age and gender between polypharmacy with fall-related fracture admission while controlling for other covariates. All statistical analyses were performed with SAS (version 9.2; SAS Institute, Cary, NC). A P value <0.05 was considered statistically significant.

For peer review only - http://bmjopen.bmj.com/site/about/guidelines.xhtml

RESULTS

Baseline characteristics of the cases and controls

The mean age for cases and controls was 78.3±7.5 years, and 73.9±6.7 years, respectively. Female dominance was seen in cases, but not in controls. Cases were more likely than controls to live in rural areas (43.7% vs. 36.6%), CCI score ≥3 (7.4% vs. 5.0%), and polypharmacy (57.8% vs. 41.5%) (Table2).

Polypharmacy and the risk factors of admission for fall-related fractures

Table 3 shows the OR of admission for fall-related fractures before and after adjusting the other variables. Compared with subjects aged 65-74 years, the adjusted odds ratio (AOR) of fall-related fractures for cases were 2.23 (95% CI=2.09-2.37) and 4.94(95% CI=4.53-5.37) in those aged 75-84 and ≥ 85 years, respectively. There were 2.19-fold (95% CI=2.06-2.33) of admission for fall-related fractures in females compared with males. Older people living in rural areas were at significantly increased risk of admission (AOR=1.31, 95% CI=1.23-1.41). Compared with a CCI score of 0, the risk of admission for fall-related fractures increased with CCI score, especially in those with scores ≥3 (AOR=1.46, 95% CI=1.28-1.66). In addition, there was a 2.22-fold of admission among patients who used polypharmacy (≥ 4 categories of medications), as opposed to those used 0 category of medication.

Age- and gender-specific for polypharmacy and admission for fall-related fractures

Table 4 shows AOR of admission for fall-related fractures in relation to category of medications according to age and gender. There was a significant interaction between

For peer review only - http://bmjopen.bmj.com/site/about/guidelines.xhtml

category of medications and age. The significant dose-gradient relationship was noted for those aged 65-74 years and 75-84 years, obviously in aged 75-84 years (β= 0.2338; P for trend< 0.0001). Compared with subjects who used 0 category of medication, subjects who used polypharmacy the AOR of admission for fall-related fractures increased to 2.46 and 2.40 for those aged 65-74 years and 75-84 years, respectively. Compared with those who used 0 category of medication, the risk of admission for fall-related fractures increased with the number of medication category in both men and women, and there was also a significant dose-gradient relationship. The AOR associated with polypharmacy was 2.16 for men, which was lower than that for women (AOR, 2.23).

For peer review only

DISCUSSION

This study used a large population-based datasets to identify the relationship of polypharmacy and fall-related fractures in older people. The major findings were as follows: age, female, living in rural areas, comobidities and category of medications were likely to have a higher risk of admission for fall-related fractures; an increasing category of medications related to fall is independently and positively associated with the risk of admission for fall-related fractures, especially in those younger older people but not obvious in those aged ≥ 85 years; the risk of admission for fall-related fractures increased with the category of medications in both men and women, and there was also a significant dose-gradient relationship.

Our findings were similar to many previous studies that risk factors for falls are increasing with age,[4, 14, 15] and polypharmacy.[3, 4, 13, 16, 17] It is likely that older people with advanced ages tended to have higher comorbidities, lack self-sufficiency, frail, and more severe form of osteoporosis, leading to an increased risk of frequent fall and more comminuted fractures.[18, 19] Ziere et al.(2006) conducted a cross-sectional study of nearly 7 thousands individuals aged ≥ 55 years and noted that falls were more common in women than in men. A systematic review which focused on the risk factors for falls in older people also showed that women at an advanced age were a predictor for falls.[20] Our study indicated that women were more likely to suffer from fall-related fractures than men. A possible explanation was that Asian women were at a high risk of osteoporosis and had a higher incidence of hip fractures than men.[21] Older people living in rural areas were likely to have a higher risk of admission for fall-related fractures. Yiannakoulias et al.[22] investigated the relationship between geographic locations and fall injuries and the study found that the surrounding rural

For peer review only - http://bmjopen.bmj.com/site/about/guidelines.xhtml

regions and smaller communities had a more moderate fall incidence. A survey of falls in Taiwan indicated that most of the fall events took place outdoors, including streets sidewalks, farmlands, and mountain areas; and those who experienced fall indoor suffered the episode mainly in the living room and bathroom.[23] A cross-sectional study found that urban residents had fewer medical diagnoses, better mobility, less pain, and fewer depressive symptoms compared with rural residents.[24] Therefore, disadvantageous indoor and outdoor environments experienced by the older people from rural Taiwan might explain the urbanization and fall relationship noted in our findings.

Huang et al.[17] found a statistically significant interaction between total number of medications and falls by different age groups (<65, ≥65 years of age) in a multi-ethnic population of type 2 diabetes patients. They found a significant increase in the risk of falls with 4-5 and 7 or more medications, and the hazard ratio (HR) were 1.45 and 2.31, respectively, compared with 0-1 prescription medications. In older patients, there was a monotonic rise in the risk of falls that became statistically significant at 6-7 (HR=1.28) or more than 7 medications (HR=1.39), compared with 0-1 prescription medications. Actually, accompanying deterioration in bone quality in older people may be the high risk group for fall-related fractures.[25, 26] In short, older people with advanced ages tend to have higher comorbidities so that the medication threshold at which fall risk begins to rise may be lower in older patients than younger patients. We divided number of medications to different group, and showed that increased risk of admission for fall-related fractures in older people who had higher use of medications, with a significant dose-gradient relationship, which further substantiates the link between polypharmacy and risk of fall-related fracture. Consequently, regular reviews of older adults' medications, minimizing the drugs

For peer review only - http://bmjopen.bmj.com/site/about/guidelines.xhtml

unless those are really necessary, may be an appropriate and costless fall prevention strategy.

This study has the following strengths. First, it was population-based and included all eligible older patients in Taiwan. Therefore, the data are highly representative and allow little room for selection bias. Second, the longitudinal data and completed reimbursement claims allow for precise medication estimation. Third, the advantage of using insurance claim datasets in clinical research is easy access to the longitudinal records for a large sample of patients. Finally, we managed to adjust, in a multivariate regression model, for a number of demographic variables and CCI scores that are considered to be risk factors for fall-related fractures. This way in research may largely reduce the play of confounding, and to point up the independent effect of polypharmacy on the risk of fall-related fractures in older patients.

Several limitations inherent with the use of administrative databases also need to be mentioned. First, we relied on the prescription records in the database, which did not provide information on how patients use medications in the real world. We also used a proxy indicator for medication use- medications purchased within the past year, not medication administration. It is possible, that in a few instances, medications were prescribed but not subsequently taken. Second, medication history was observed for only 1 year before the fall-related fractures. This may underestimate the medication-associated risk of fall-related fractures. Third, only falls related to hospitalization and hospital treatment are presented. Falls which were treated in clinics, self-treated or that did not result in an injury are not included. Finally, the other potential risk factors of fall, such as environmental factors and living arrangement were not available from the NHI datasets, so that we were unable to take

For peer review only - http://bmjopen.bmj.com/site/about/guidelines.xhtml

these potential confounders in to consideration.

In summary, this population-based study provides further evidence that polypharmacy may increase fall-related fractures risk in older people, especially in the younger age groups. To further reduce the incidence of fall-related fractures caused by polypharmacy, clinicians and researchers must consider an algorithm that may effectively identify inappropriate polypharmacy prescriptions, and more attention should be paid to the younger older people.

For peer review only

For peer review only - http://bmjopen.bmj.com/site/about/guidelines.xhtml

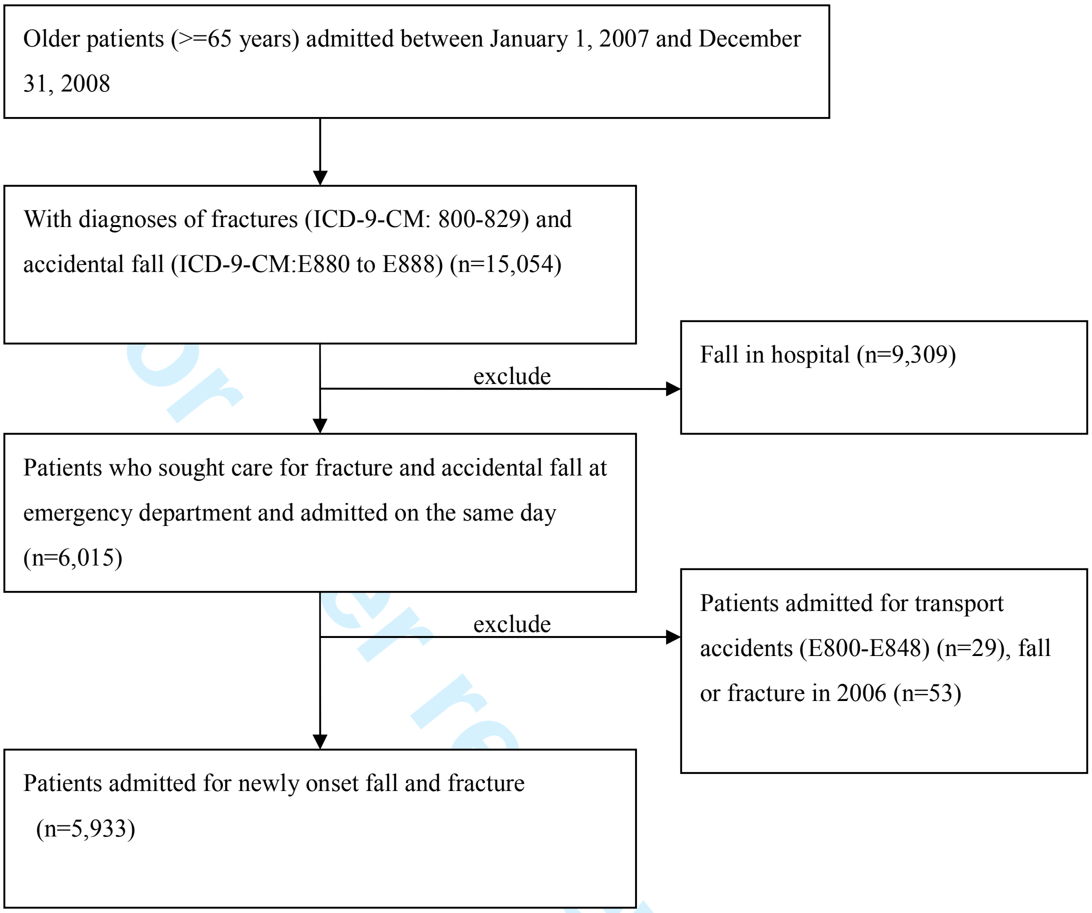

Figure 1 Flow chart of cases

For peer review only - http://bmjopen.bmj.com/site/about/guidelines.xhtml

Table 1 Medications related to fall calculated with WHO ATC classification system

Category of medications related to fall	ATC index
**Allmentary tract and metabolism**	
Drugs for functional gastrointestinal disorders	A03
Blood glucose lowering drugs and insulin	A10A, A10B
Vitamins	A11
**Blood and blood forming organs**	
Antithrombotic agents	B01A
**Cardiovascular system**	
Cardiac glycosides and nitrates	C01A, C01DA
Antiarrhythmics	C01B
Antihypertensives	C02
Diuretics	C03
**Musculo-skeletal system**	
Non-steroidal anti-inflammatory drugs (NSAIDs)	M01A
**Nervous system**	
Opioids	N02A
Antiepileptics	N03
Antiparkinson drugs	N04
Antipsychotics	N05A
Anxiolytics	N05B
Hypnotics and sedatives	N05C
Antidepressants	N06A

ATC, Anatomical Therapeutic Chemical

For peer review only

For peer review only - http://bmjopen.bmj.com/site/about/guidelines.xhtml

Table 2 Comparisons of sociodemographics and medications between cases and controls

	Cases (n=5,933)		Controls (n=29,665)		P value
	n	%	n	%	
Age, mean±SD	78.3	±7.5	73.9	±6.7	<0.0001
Age group, y					<0.0001
65-74	1,956	33.0	17,082	57.6	
75-84	2,671	45.0	10,270	34.6	
≥85	1,306	22.0	2,313	7.8	
Gender					<0.0001
Male	1,833	30.9	14,678	49.5	
Female	4,100	69.1	14,987	50.5	
Urbanization					<0.0001
Urban	1,930	32.5	10,934	36.9	
Satellite	1,408	23.7	7,884	26.6	
Rural	2,595	43.7	10,847	36.6	
CCI score					<0.0001
0	1,869	31.5	12,398	41.8	
1	2,595	43.7	11,982	40.4	
2	1,030	17.4	3,810	12.8	
≥3	439	7.4	1,475	5.0	
Category of medications					
0	348	5.9	3,464	11.7	<0.0001
1	523	8.8	3,984	13.4	
2	775	13.1	4,913	16.6	
3	857	14.4	4,995	16.8	
>=4	3,430	57.8	12,309	41.5	

CCI, Charlson comorbidity index.

For peer review only

Table 3 Crude and adjusted odds ratios and 95% confidence intervals of admission for fall-related fractures in relation to socio-demographics and medications

	Crude estimate		Adjusted estimate	
	OR	95%CI	OR	95%CI
Age group, y				
65-74	1.00	reference	1.00	reference
75-84	2.27	2.13-2.42	2.23	2.09-2.37
≥85	4.93	4.54-5.36	4.94	4.53-5.37
			β= 0.7953; P for trend< 0.0001	
Gender				
Male	1.00	reference	1.00	reference
Female	2.19	2.06-2.33	2.19	2.06-2.33
Urbanization				
Urban	1.00	reference	1.00	reference
Satellite	1.01	0.94-1.09	1.03	0.96-1.12
Rural	1.36	1.27-1.45	1.31	1.23-1.41
			β= 0.1370; P for trend< 0.0001	
CCI				
0	1.00	reference	1.00	reference
1	1.44	1.35-1.53	1.08	1.00-1.16
2	1.79	1.65-1.95	1.26	1.15-1.39
≥3	1.97	1.76-2.22	1.46	1.28-1.66
			β= 0.1008; P for trend< 0.0001	
Category of medications				
0	1.00	reference	1.00	reference
1	1.31	1.13-1.51	1.29	1.11-1.49
2	1.57	1.37-1.80	1.46	1.33-1.60
3	1.71	1.50-1.95	1.51	1.31-1.73
≥4	2.77	2.47-3.12	2.22	1.95-2.52
			β= 0.1943; P for trend< 0.0001	

CCI, Charlson comorbidity index; OR, odds ratio; CI, confidence interval.

For peer review only - http://bmjopen.bmj.com/site/about/guidelines.xhtml

Table 4 Covariate adjusted odds ratios and 95% confidence intervals of admission for fall-related fractures in relation to category of medications according to gender and age

Category of medications	Gender				Age (year)					
	Men		Women		65-74		75-84		≥85	
	AOR	95% CI	AOR	95% CI	AOR	95% CI	AOR	95% CI	AOR	95% CI
0	1.00	reference	1.00	reference	1.00	reference	1.00	reference	1.00	reference
1	1.31	1.03-1.67	1.26	1.05-1.52	1.49	1.17-1.90	1.11	0.86-1.43	1.34	1.00-1.81
2	1.39	1.10-1.75	1.48	1.24-1.77	1.66	1.32-2.09	1.55	1.23-1.96	1.12	0.84-1.50
3	1.55	1.23-1.96	1.47	1.24-1.75	1.62	1.28-2.04	1.71	1.36-2.14	1.13	0.84-1.51
≥4	2.16	1.75-2.68	2.23	1.90-2.62	2.46	1.98-3.05	2.40	1.95-2.97	1.69	1.30-2.20
	β= 0.1873; P for trend< 0.0001		β= 0.1962; P for trend< 0.0001		β= 0.1962; P for trend< 0.0001		β= 0.2338; P for trend< 0.0001		β= 0.1168; P for trend< 0.0001	

AOR, adjusted odds ratio; adjustment for age, urbanization, and Charlson index. CI, confidence interval.

For peer review only - http://bmjopen.bmj.com/site/about/guidelines.xhtml

Acknowledgements The authors would like to thank Taipei Veterans General Hospital to sponsor this study.

Contributors HH analyzed the data and wrote the manuscript. CY, TJ, TP and KY designed the study and revised the manuscript.

Funding This work was supported by a grant from Taipei Veterans General Hospital (V100C-211). The organizations that sponsored this study were not involved in the design of this study, data-collection, data processing or manuscript preparation.

Competing interests None declared

For peer review only

For peer review only - http://bmjopen.bmj.com/site/about/guidelines.xhtml

REFERENCES

1. Ministry of the Interior, Taiwan, 2010. *Resident population by 5-year, 10-year age group*. http://sowf.moi.gov.tw/stat/year/list.htm
2. Rao SS. Prevention of falls in older patients. *Am Fam Physician* 2005; 72: 81-8.
3. Ziere G, Dieleman JP, Hofman A*, et al.* Polypharmacy and falls in the middle age and elderly population. *Br J Clin Pharmacol* 2006; 61: 218-23.
4. Corsinovi L, Bo M, Ricauda Aimonino N*, et al.* Predictors of falls and hospitalization outcomes in elderly patients admitted to an acute geriatric unit. *Arch Gerontol Geriatr* 2009; 49: 142-5.
5. Vassallo M, Sharma JC,Allen SC. Characteristics of single fallers and recurrent fallers among hospital in-patients. *Gerontology* 2002; 48: 147-50.
6. Kannus P, Sievanen H, Palvanen M*, et al.* Prevention of falls and consequent injuries in elderly people. *Lancet* 2005; 366: 1885-93.
7. Hartikainen S, Lonnroos E, Louhivuori K. Medication as a risk factor for falls: critical systematic review. *J Gerontol A Biol Sci Med Sci* 2007; 62: 1172-81.
8. Lai SW, Liao KF, Liao CC*, et al.* Polypharmacy correlates with increased risk for hip fracture in the elderly: a population-based study. *Medicine* 2010; 89: 295-9.
9. French DD, Campbell R, Spehar A*, et al.* Outpatient medications and hip fractures in the US: a national veterans study. *Drugs Aging* 2005; 22: 877-85.
10. Lu JFR, Hsiao WC. Does universal health insurance make health care unaffordable? Lessons from Taiwan. *Health Affairs (Millwood)* 2003; 22: 77-88.
11. Directorate-General Budget , 1993. National Statistics of Regional Standard Classification Data, Taipei, Taiwan.
12. Charlson ME, Pompei P, Ales KL*, et al.* A new method of classifying prognostic

For peer review only - http://bmjopen.bmj.com/site/about/guidelines.xhtml

comorbidity in longitudinal studies: development and validation. *J Chronic Dis* 1987; 40: 373-83.

13. Baranzini F, Poloni N, Diurni M*, et al.* Polypharmacy and psychotropic drugs as risk factors for falls in long-term care setting for elderly patients in Lombardy. *Recenti Prog Med* 2009; 100: 9-16.

14. Kojima T, Akishita M, Nakamura T*, et al.* Polypharmacy as a risk for fall occurrence in geriatric outpatients. *Geriatr Gerontol Int* 2012; 12: 425-30.

15. Krauss MJ, Nguyen SL, Dunagan WC*, et al.* Circumstances of patient falls and injuries in 9 hospitals in a midwestern healthcare system. *Infection control and hospital epidemiology : the official journal of the Society of Hospital Epidemiologists of America* 2007; 28: 544-50.

16. Baranzini F, Diurni M, Ceccon F*, et al.* Fall-related injuries in a nursing home setting: is polypharmacy a risk factor? *BMC Health Serv Res* 2009; 9: 228.

17. Huang ES, Karter AJ, Danielson KK*, et al.* The association between the number of prescription medications and incident falls in a multi-ethnic population of adult type-2 diabetes patients: the diabetes and aging study. *J Gen Intern Med* 2010; 25: 141-6.

18. Chen JS, Simpson JM, March LM*, et al.* Risk factors for fracture following a fall among older people in residential care facilities in Australia. *J Am Geriatr Soc* 2008; 56: 2020-6.

19. Martinez AA, Cuenca J, Panisello JJ*, et al.* Changes in the morphology of hip fractures within a 10-year period. *Journal of bone and mineral metabolism* 2001; 19: 378-81.

20. Gama ZA,Gomez-Conesa A. [Risk factors for falls in the elderly: systematic review. *Rev Saude Publica* 2008; 42: 946-56.

21. Shao CJ, Hsieh YH, Tsai CH*, et al.* A nationwide seven-year trend of hip fractures

For peer review only - http://bmjopen.bmj.com/site/about/guidelines.xhtml

in the elderly population of Taiwan. *Bone* 2009; 44: 125-9.

22. Yiannakoulias N, Rowe BH, Svenson LW*, et al.* Zones of prevention: the geography of fall injuries in the elderly. *Social science & medicine (1982)* 2003; 57: 2065-73.

23. Chang HJ, Chang YF, Yang YC*, et al.* Epidemiological survey of falls and associated risk factors in old females in an aging community. *Taiwan Geriatrics & Gerontology* 2010; 5: 50-61.

24. Sigurdardottir AK, Arnadottir SA,Gunnarsdottir ED. Medication use among community-dwelling older Icelanders. Population-based study in urban and rural areas. *Laeknabladid* 2011; 97: 675-80.

25. Taylor AJ, Gary LC, Arora T*, et al.* Clinical and demographic factors associated with fractures among older Americans. *Osteoporos Int* 2011; 22: 1263-74.

26. Watson WL, Mitchell R. Conflicting trends in fall-related injury hospitalisations among older people: variations by injury type. *Osteoporos Int* 2011; 22: 2623-31.

For peer review only

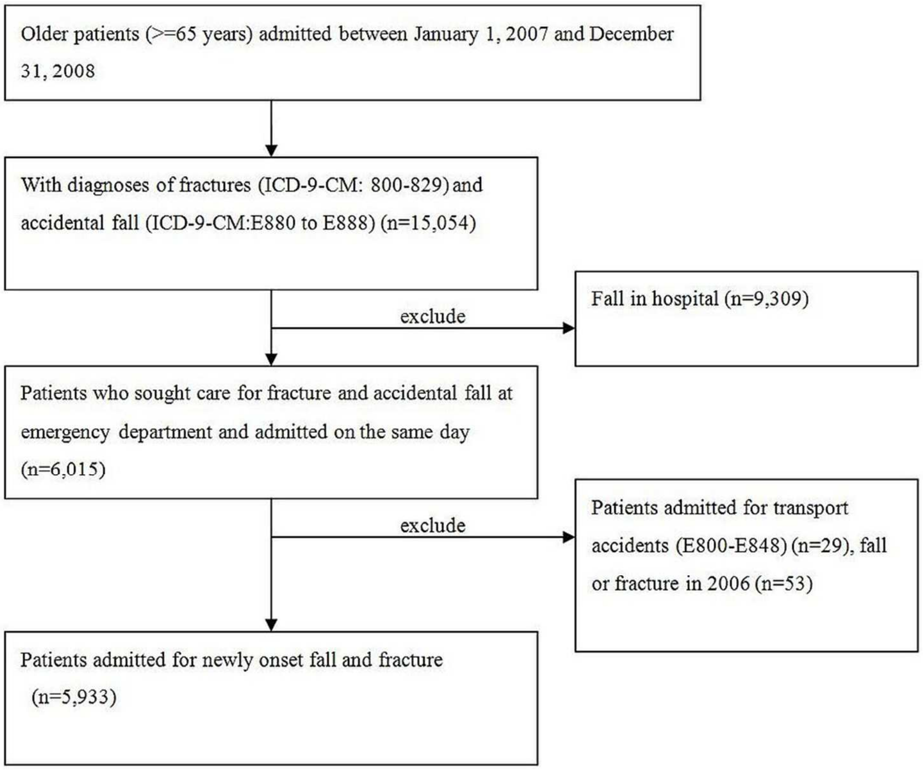

90x72mm (300 x 300 DPI)

ew only

STROBE 2007 (v4) Statement—Checklist of items that should be included in reports of *case-control studies*

Section/Topic	Item #	Recommendation	Reported on page #
**Title and abstract**	1	(*a*) Indicate the study's design with a commonly used term in the title or the abstract	3
		(*b*) Provide in the abstract an informative and balanced summary of what was done and what was found	3
**Introduction**			
Background/rationale	2	Explain the scientific background and rationale for the investigation being reported	6
Objectives	3	State specific objectives, including any prespecified hypotheses	6
**Methods**			
Study design	4	Present key elements of study design early in the paper	7
Setting	5	Describe the setting, locations, and relevant dates, including periods of recruitment, exposure, follow-up, and data collection	7
Participants	6	(*a*) Give the eligibility criteria, and the sources and methods of case ascertainment and control selection. Give the rationale for the choice of cases and controls	7-8
		(*b*) For matched studies, give matching criteria and the number of controls per case	8
Variables	7	Clearly define all outcomes, exposures, predictors, potential confounders, and effect modifiers. Give diagnostic criteria, if applicable	8
Data sources/ measurement	8*	For each variable of interest, give sources of data and details of methods of assessment (measurement). Describe comparability of assessment methods if there is more than one group	7-9
Bias	9	Describe any efforts to address potential sources of bias	8-9
Study size	10	Explain how the study size was arrived at	7
Quantitative variables	11	Explain how quantitative variables were handled in the analyses. If applicable, describe which groupings were chosen and why	8-9
Statistical methods	12	(*a*) Describe all statistical methods, including those used to control for confounding	9
		(*b*) Describe any methods used to examine subgroups and interactions	9
		(*c*) Explain how missing data were addressed	9
		(*d*) If applicable, explain how matching of cases and controls was addressed	8-9
		(*e*) Describe any sensitivity analyses	9

For peer review only

**Results**			
Participants	13*	(a) Report numbers of individuals at each stage of study—eg numbers potentially eligible, examined for eligibility, confirmed eligible, included in the study, completing follow-up, and analysed	10
		(b) Give reasons for non-participation at each stage	10
		(c) Consider use of a flow diagram	16, figure 1
Descriptive data	14*	(a) Give characteristics of study participants (eg demographic, clinical, social) and information on exposures and potential confounders	10
		(b) Indicate number of participants with missing data for each variable of interest	10
Outcome data	15*	Report numbers in each exposure category, or summary measures of exposure	10-11
Main results	16	(*a*) Give unadjusted estimates and, if applicable, confounder-adjusted estimates and their precision (eg, 95% confidence interval). Make clear which confounders were adjusted for and why they were included	10-11
		(*b*) Report category boundaries when continuous variables were categorized	10-11
		(*c*) If relevant, consider translating estimates of relative risk into absolute risk for a meaningful time period	10-11
Other analyses	17	Report other analyses done—eg analyses of subgroups and interactions, and sensitivity analyses	10-11
**Discussion**			
Key results	18	Summarise key results with reference to study objectives	12
Limitations	19	Discuss limitations of the study, taking into account sources of potential bias or imprecision. Discuss both direction and magnitude of any potential bias	14
Interpretation	20	Give a cautious overall interpretation of results considering objectives, limitations, multiplicity of analyses, results from similar studies, and other relevant evidence	15
Generalisability	21	Discuss the generalisability (external validity) of the study results	15
**Other information**			
Funding	22	Give the source of funding and the role of the funders for the present study and, if applicable, for the original study on which the present article is based	21

*Give information separately for cases and controls in case-control studies and, if applicable, for exposed and unexposed groups in cohort and cross-sectional studies.

Note: An Explanation and Elaboration article discusses each checklist item and gives methodological background and published examples of transparent reporting. The STROBE checklist is best used in conjunction with this article (freely available on the Web sites of PLoS Medicine at http://www.plosmedicine.org/, Annals of Internal Medicine at http://www.annals.org/, and Epidemiology at http://www.epidem.com/). Information on the STROBE Initiative is available at www.strobe-statement.org.

For peer review only

For peer review only
